# Overexpression of the Wheat *TaPsb28* Gene Enhances Drought Tolerance in Transgenic *Arabidopsis*

**DOI:** 10.3390/ijms24065226

**Published:** 2023-03-09

**Authors:** Yuexia Wang, Menghan Zhang, Xiaoyan Li, Ruixiang Zhou, Xinyu Xue, Jing Zhang, Nana Liu, Ruili Xue, Xueli Qi

**Affiliations:** 1 College of Life Sciences, Henan Agricultural University, Zhengzhou 450002, China; 2 Department of Biological Science, Purdue University, West Lafayette, IN 47907, USA; 3 Institute of Crops Molecular Breeding, Henan Academy of Agricultural Sciences, Zhengzhou 450002, China; 4 The Shennong Laboratory, Zhengzhou 450002, China

**Keywords:** wheat, drought stress, *TaPsb28* gene, overexpression, anthocyanidins, stomatal closure

## Abstract

Psb28 is a soluble protein in the photosystem II (PSII) complex, but its role in the drought stress response of wheat remains unclear. Here, we functionally characterized the *TaPsb28* gene, which positively regulates drought tolerance in wheat. When the full-length 546-bp *TaPsb28* cDNA was transferred into *Arabidopsis thaliana*, it was located in the guard cell chloroplast around the stroma. Overexpression of *TaPsb28* conferred drought tolerance, as exhibited by the increases in the survival rate. Transgenic plants maintained lower MDA content and higher chlorophyll content by inducing chlorophyll synthase (*ChlG*) gene transcription. The content of abscisic acid (ABA) and zeatin increased significantly in wild-type (WT) plants under drought stress, and the transcriptional expression levels of *RD22*, dihydroflavonol 4-reductase (*DFR*) and anthocyanin reductase (*ANR*) genes were induced, thus enhancing the contents of endogenous cyanidin, delphinidin, and proanthocyanidins. However, in transgenic plants, although anthocyanins were further aggregated, the ABA increase was inhibited, zeatin was restored to the control level under drought stress, and stomatal closure was promoted. These findings indicate ABA and zeatin have opposite synergistic effects in the process of drought tolerance caused by *TaPsb28* because only after the effect of zeatin is alleviated can ABA better play its role in promoting anthocyanin accumulation and stomatal closure, thus enhancing the drought tolerance of transgenic plants. The results suggest that overexpression of *TaPsb28* exerts a positive role in the drought response by influencing the functional metabolism of endogenous hormones. The understanding acquired through the research laid a foundation for further in-depth investigation of the function of *TaPsb28* in drought resistance in wheat, especially its relationship with anthocyanidin accumulation.

## 1. Introduction

Wheat (*Triticum aestivum* L.) is one of the world’s most important crops, and drought is one of the most common environmental threats to plant growth and productivity. It is of great significance to fully explore the drought tolerance potential of wheat and improve yield by clarifying the drought tolerance mechanism of wheat [1].

Chloroplasts are the main site of photosynthesis and the center of plant growth and development and abiotic stress response. They are sensitive to stress and are important biosensors for plants to respond to environmental turbulence [2,3], and they are the main source of reactive oxygen species (ROS) [4]. Psb28 is an extrinsic protein of photosystem II (PSII), which is conserved among photosynthetic organisms from cyanobacteria to higher plants. Psb28 is one of the assembly factors of PSII and plays an important role in the repair of PSII under stress conditions [5]. Transcriptome analysis showed that *psb28* gene transcription was induced through circRNA-miRNA-mRNA regulation under drought stress [6]. Its transcriptional induction by exogenous 5-aminolevulinic acid was also closely related to drought tolerance in different wheat cultivars [7]. In addition, the protein and/or transcript levels of Psb28 have also been reported to decrease in drought-stressed *Nostoc flagelliforme* [8], *Panicum miliaceum* [9], and maize [10]. *Psb28* was defined as one of the most important stress–responsive genes, because its upregulation was most likely to associate with the alleviation of the damage to plant chloroplast ultrastructure and photosynthesis [7]. In rice, leaves exhibited a light-green phenotype after the *Psb28* gene was inactivated by T-DNA insertion, indicating that it is required for normal growth and pigmentation and that this protein may contribute to stabilizing PSII activity [11]. However, until now, the physiological regulation mechanisms of *Psb28* remain poorly understood.

Photosynthetic pigments, as chloroplast, play an important role in photosynthesis by capturing light energy and inducing electron transfer, while water stress can restrain chlorophyll synthesis and reduce the photosynthetic pigment content, thus decreasing the photosynthetic efficiency in leaves [12]. Chlorophyll synthase (ChlG) is the last enzyme in the chlorophyll biosynthesis pathway, which plays an important role in the coordination between the entire metabolic pathway of chlorophyll synthesis and the synthesis of chlorophyll-binding protein [13], as well as the reutilization of chlorophyllide during chlorophyll turnover under environmental stress [14,15]. Anthocyanins are present in the vacuoles of plant cells, and *Arabidopsis* plants accumulated anthocyanins and exhibited increasing tolerance to drought [16]. The biosynthesis of anthocyanin involves a variety of enzymes and is a complex process influenced and regulated by environmental conditions. Dihydroflavonol 4-reductase (DFR) and anthocyanidin reductase (ANR) are two key enzymes in the anthocyanin biosynthesis pathway [17]. However, there is no evidence of a possible association between *TaPsb28* gene function and photosynthetic pigment synthesis.

Under drought stress, plants control their transpiration rate to reduce water loss through stomatal closure, so leaf stomatal character is one of the important indicators with which to explore adaptation [18]. The plant endogenous hormone abscisic acid (ABA) is the most important chemical signal regulating stomatal closure. The smaller stomatal opening triggered by ABA improved the water use efficiency and drought resistance under drought conditions [19]. In addition to ABA, endogenous zeatin also plays an important role in plant development and response to abiotic stress [20]. The promoting action of spermidine on the grain-filling characteristics of wheat under drought stress was notably related to an increase in endogenous zeatin content [21]. However, the roles of ABA and zeatin in regulating drought tolerance when the *TaPsb28* gene is overexpressed is still not well understood.

Our previous studies revealed that the wheat *TaPsb28* gene was upregulated under drought stress [7], suggesting that it may be involved in the drought response. In this study, to characterize the role of *TaPsb28* in response to drought stress, it was overexpressed in transgenic *Arabidopsis* and the following physiological and molecular responses to drought stress were analysed. We found that *TaPsb28* lies in the guard cell chloroplast around the stoma of transgenic *Arabidopsis* plant leaves. Overexpression of *TaPsb28* confers tolerance to drought stress, with increased survival and reduced leaf water loss compared with wild-type plants. Subsequently, we identified an oppositely synergistic effect of ABA and zeatin in the process of drought tolerance caused by *TaPsb28* because only after the effect of zeatin is alleviated can ABA better play its role in inhibiting chlorophyll content decrease and promoting anthocyanin aggregation and stomatal closure by regulating relative gene transcription. These results indicate that *TaPsb28* exerts a positive role in the drought response by influencing the functional metabolism of endogenous hormones.

## 2. Results and Discussion

### 2.1. Sequence Analysis of the Wheat TaPsb28 Gene

**Figure 1:**
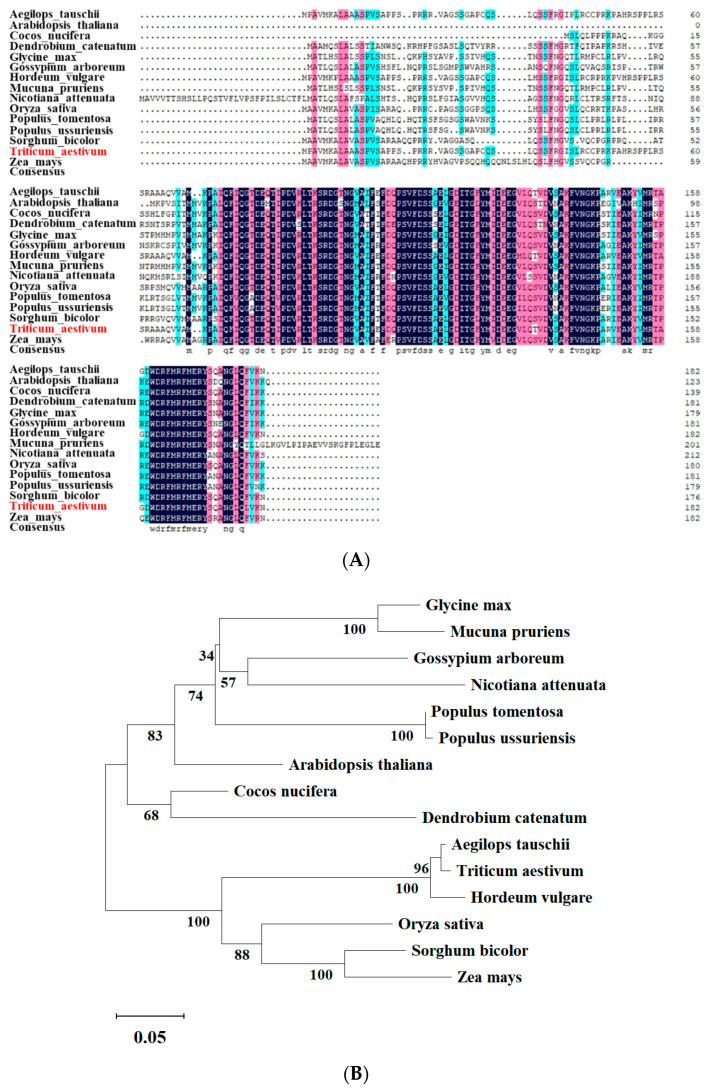
Multiple sequence alignment (**A**) and the phylogenetic tree (**B**) of the plant Psb28 family proteins. The wheat *TaPsb28* gene sequences were downloaded from the WheatlS database (https://urgi.versailles.inra.fr/wheatis, accessed on 10 October 2018), while homologous protein sequences were retrieved from the National Center for Biotechnology Information (NCBI). The different color shading of the alignment represents different degrees of conservation among sequences, and the dark blue shading indicates identical amino acids.

As shown in Figure 1, the full-length coding sequence (CDS) of *TaPsb28* was retrieved from WheatIS. *TaPsb28* encoded a protein of 182 amino acids with a predicted mass of 19.8 kDa and a theoretical PI of 10.15. A phylogenetic tree of amino acid sequences was constructed using *TaPsb28* and fourteen Psb28 orthologues of different plant species (Figure 1B). The amino acid of *TaPsb28* shared the highest homology with *Aegilops tauschii AetPsb28* (96%), followed by *Hordeum vulgare HuPsb28*, *Oryza sativa OsPsb28*, *Sorghum bicolor SbPsb28*, and *Zea mays ZmPsb28*.

### 2.2. Expression Pattern under Drought Stress and Subcellular Localization of Wheat TaPsb28

**Figure 2:**
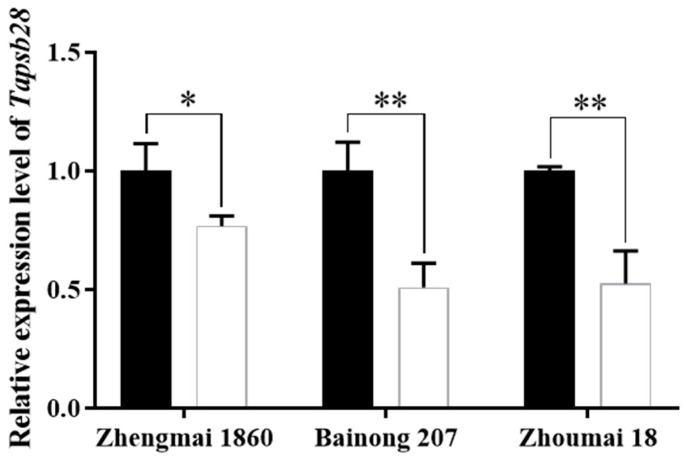
Relative expression of *TaPsb28* in wheat leaves under drought stress. The black column represents the seedlings with normal watering, while the white column represents the seedlings after 7 d of drought treatment by ceasing watering. Three wheat cultivars, Zhengmai 1860, BaiNong 207, and Zhoumai18, were used as plant materials when two leaves were fully expanded. Data are expressed as the means ± SDs (*n* = 3). “**” and “*” indicate statistically significant differences between the CK and drought treatments at *p* < 0.01 and 0.05, respectively.

Psb28 is one of the assembly factors of PSII and plays an important role in the recovery of PSII under environmental stress [5]. To investigate the response of wheat *TaPsb28* gene transcription under drought stress, we exposed seedlings of three wheat cultivars, Zhengmai 1860, Bainong 207, and Zhoumai 18, to 7-day drought stress. As shown in Figure 2, the transcription of *TaPsb28* in the three tested wheat seedlings all decreased significantly by 23%, 49%, and 47%, respectively (*p* < 0.05), suggesting that the transcription of *psb28* is associated with the resistance of wheat to drought stress study [7].

**Figure 3:**
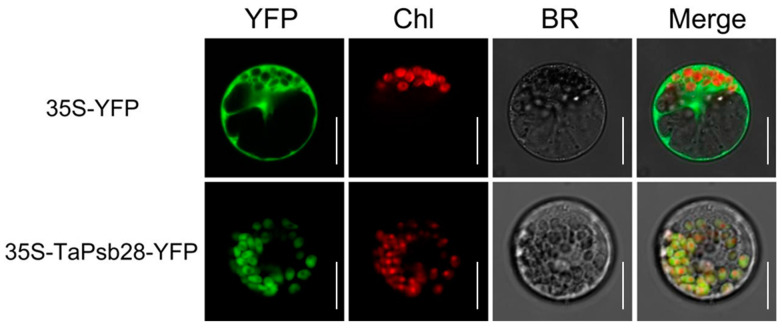
Subcellular localization of *TaPsb28*-YFP in maize mesophyll protoplasts. YFP: yellow fluorescent protein; Chl: chloroplast self-luminescence; BR: bright field. Bars = 10 μm.

To observe the acting position of *TaPsb28*, we carried out transient expression investigations with the pYFPLT-*TaPsb28* construct. As shown in Figure 3, the autofluorescence of chloroplasts was red, as observed by laser scanning confocal microscopy. When the 35S-YFP empty vector was transformed into maize mesophyll protoplasts, the green fluorescent signal was detected in the whole cell in addition to the vacuole and chloroplast. Comparatively, in *TaPsb28*-YFP fusion-expressing cells, green fluorescence was only detectable in the chloroplast stroma, which indicated that *TaPsb28* located in the chloroplast stroma.

### 2.3. Generation of TaPsb28-Overexpressing Transgenic Arabidopsis Plants and Subcellular Localization

**Figure 4:**
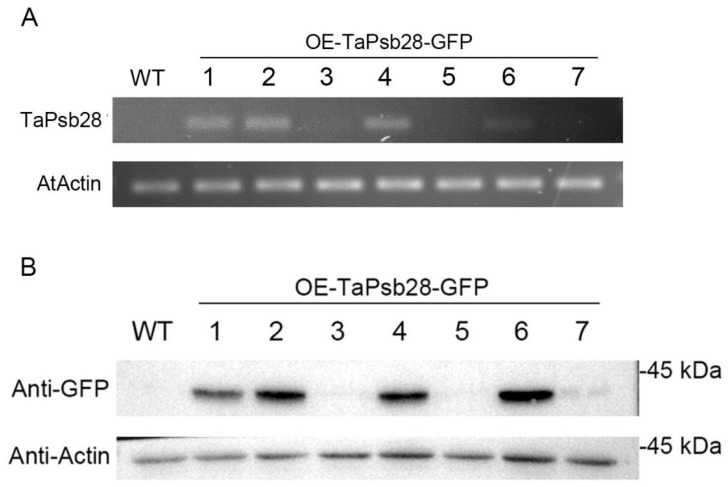
RT–PCR (**A**) and Western blot (**B**) identification of T2-generation OE-*TaPsb28* plants.

To evaluate whether *TaPsb28* overexpression can improve tolerance against drought stress, we constructed a full coding sequence of *TaPsb28* in pCAMBIA1302-*TaPsb28* and transformed it into wild-type *A. thaliana* (Columbia-0). After screening the homozygous T2 generations, seven homozygous lines were obtained and named OE-*TaPsb28*/WT-1 to OE-*TaPsb28*/WT-7. Thereafter, the presence of *TaPsb28* was identified by both RT–PCR (Figure 4A) and Western blot assays (Figure 4B), and four positive plants were obtained: L1, L2, L4, and L6. Thereafter, three overexpressing lines (L2, L4, and L6) were used for subsequent experiments because they exhibited comparatively higher expression levels of *TaPsb28*. The line L1 was discarded because only a weak signal was observed by the Western blot assay.

Drought stress is one of the main limiting factors of global agricultural production due to the complexity of water-restricted environments and climate change [1]. It is of increasing importance to develop crop plants with improved drought stress tolerance using traditional breeding or transgenic approaches. Overexpression of the functional genes in a model plant such as *A. thaliana* has been demonstrated to be promising in elucidating its function in the drought stress resistance of wheat. For example, overexpression of TaWRKY53 can enhance drought tolerance in transgenic *Arabidopsis* [22]. Overexpression of paralogues of the wheat *TaEXPA8* gene improves low-temperature tolerance in *Arabidopsis* [23]. Herein, a new *TaPsb28* gene cloned from wheat was overexpressed in *Arabidopsis* to reveal its possible function in response to drought.

**Figure 5:**
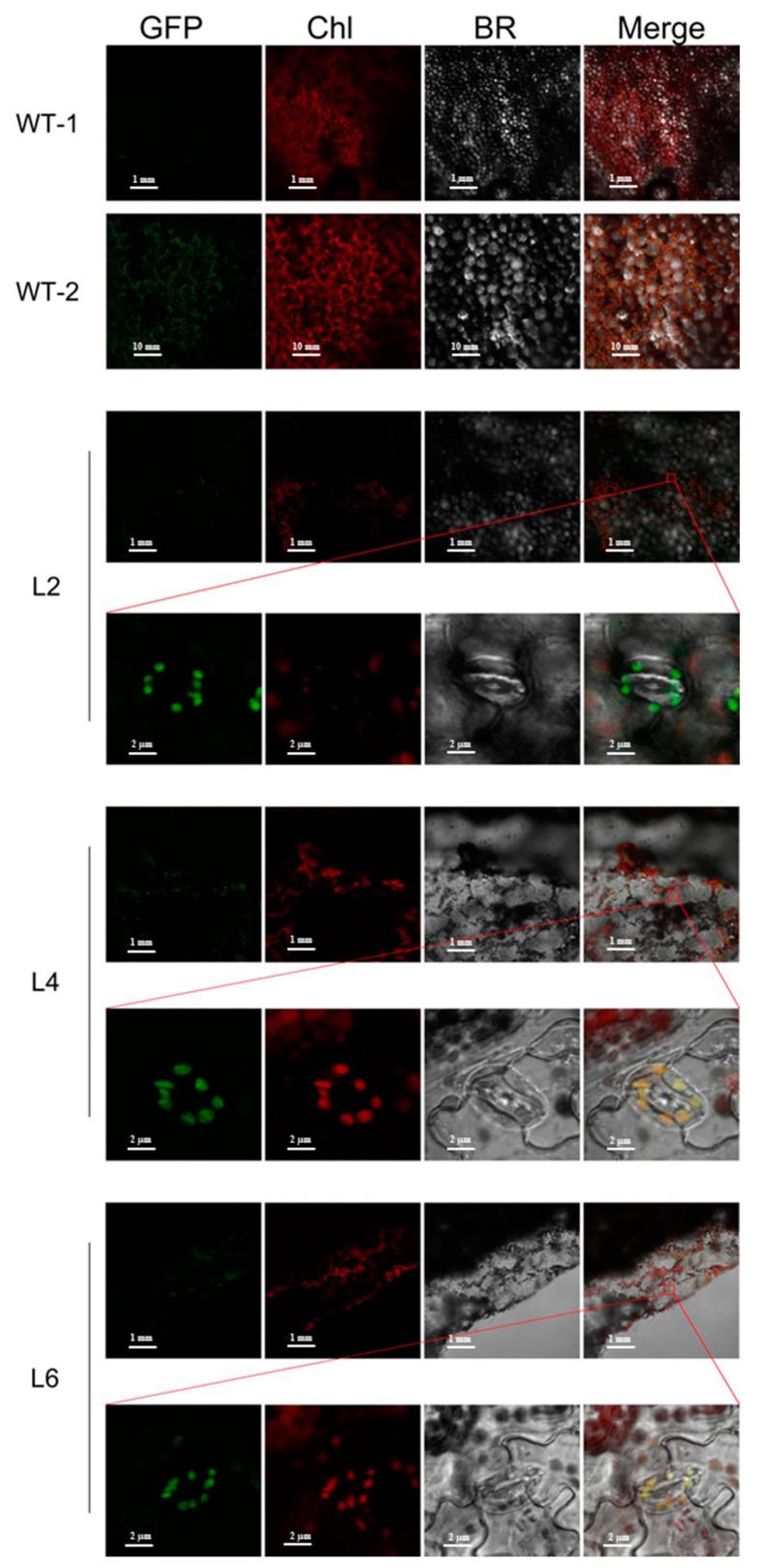
Subcellular localization of OE-*TaPsb28* in transgenic *Arabidopsis* lines. GFP: yellow fluorescent protein; Chl: chloroplast self-luminescence; BR: bright field.

The L2-, L4- and L6-overexpressing transgenic plant lines were used to further subcellularly localize *TaPsb28* in *Arabidopsis*. As shown in Figure 5, the GFP green fluorescence signal could only be observed in the guard cell chloroplast around the stoma of the three transgenic plants, indicating that *TaPsb28* located in the chloroplast but specific to guard cells around the stoma. Guard cells play an important role in H_2_O_2_ or ABA signaling stomatal closure after drought conditions are initiated [16,24]. It is known that the guard cell chloroplast is the site of carbon assimilation for photosynthesis. It is a process in which green plants use the synthesizing forces (ATP and NADPH) formed in the light reaction to synthesize sugars by consuming CO_2_ [25]. PSII is a multisubunit protein complex distributed on the inner side of chloroplast thylakoid membranes. Therefore, it can be hypothesized that in wheat, *Psb28* is expressed in the chloroplast stroma of cells and acts as one of the assembly factors of PSII, which may be involved in the process of PSII assembly and repair under drought stress [26].

### 2.4. Overexpression of TaPsb28 Enhanced the Drought Tolerance of Transgenic Arabidopsis Seedlings

**Figure 6:**
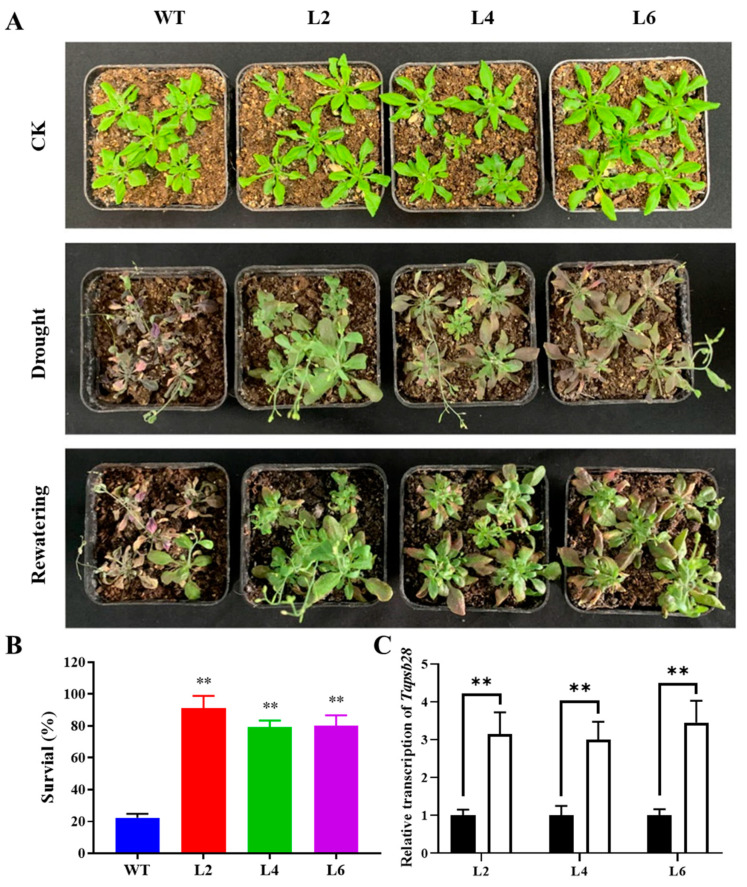
Phenotypic effects of *TaPsb28* overexpression in *Arabidopsis* seedlings exposed to drought treatment. (**A**) Growth status of *Arabidopsis* lines under different treatments; (**B**) Comparison of survival rate of overexpression lines after 5 days of rewatering followed by 14 days of drought stress by withholding irrigation; (**C**) qRT–PCR analysis of *TaPsb28* gene transcription in the transgenic plants. The black column represents the plants treated by normal watering, while the white column represents the plants subjected to drought stress by withholding irrigation for 14 days. Data are the means ± SDs (*n* = 3). “**” indicates statistically significant differences between the transgenic and WT plants in (**B**) or between drought and CK treatment in (**C**) at *p* < 0.01.

Two-week-old wild-type and L2, L4 and L6 lines of transgenic *Arabidopsis* were subjected to drought stress by water shortage to observe the phenotypic effects of drought on seedlings. We found no significant difference in morphology between wild-type and transgenic plants in normal culture without drought stress (Figure 6). However, after 14 d of drought treatment, the WT seedlings suffered more severe chlorosis and died from bottom to top. The predominant leaves of the three overexpression lines were still green without significant withering, although some leaves were yellow. After 5 d of rewatering following drought stress, the survival rate of WT was only 22%, while the survival rates of the overexpression lines were significantly higher, at 91%, 79%, and 80% for L2, L4, and L6, respectively. The results suggested that the three overexpression lines have comparatively higher drought tolerance than WT. 

### 2.5. Overexpression of TaPsb28 Decreased the Stomatal Aperture and Water Loss under Drought Stress

**Figure 7:**
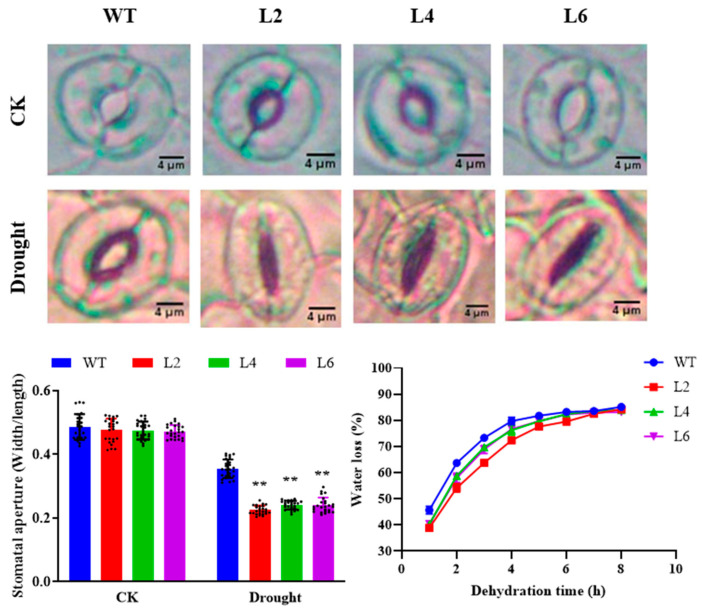
Stomatal aperture and water loss rate of *TaPsb28*-overexpressing *Arabidopsis* seedlings in response to drought stress. The images of stomatal phenotype were obtained by a microscope. Data are expressed as the means ± SDs (*n* = 25). “**” indicates statistically significant differences between the plants subjected to the drought and CK treatments at *p* < 0.01.

To observe the responses of stomatal apertures to drought stress, the leaves from WT and transgenic *Arabidopsis* seedlings were dried in vitro for 2 h and then observed by a microscope. As shown in Figure 7, all the plants showed obvious closure when subjected to drought stress. The stomatal aperture of the overexpression lines was significantly higher than that of the WT, as described by the ratio values of width to length.

**Figure 8:**
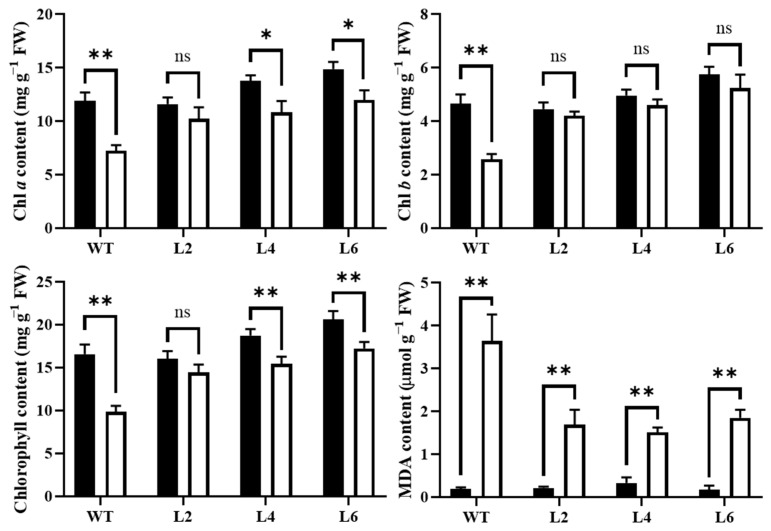
Chlorophyll and MDA contents of *TaPsb28*-overexpressing *Arabidopsis* seedlings exposed to drought treatment. The black column represents the plants treated by normal watering, while the white column represents the plants subjected to drought stress by withholding irrigation for 14 days. Data are expressed as the means ± SDs (*n* = 3). “**” and “*” indicate statistically significant differences between the CK and drought treatments at *p* < 0.01 and 0.05, respectively, while “ns” represents nonsignificant differences between the two treatments at the level of *p* < 0.05.

Three-week-old WT and transgenic *Arabidopsis* seedlings were used to evaluate their water loss rate under air-drying conditions at 21 °C. As shown in Figure 8, the water loss rate of the overexpression lines was significantly lower than that of the WT at each time point within 5 h. However, the difference declined with further air drying, and no significant difference could be observed between the transgenic plants and WT. The results indicated that the water retention capacity in the leaves of the overexpression lines was higher than that of the WT in the initial drought treatment.

### 2.6. Overexpression of TaPsb28 Increased the Chlorophyll Content and Decreased the MDA Content under Drought Stress

Drought stress obviously decreased the chlorophyll content of *Arabidopsis* WT seedlings, including the total chlorophyll, chlorophyll *a*, and chlorophyll *b* contents (Figure 8). The chlorophyll contents in *TaPsb28*-overexpression lines also declined in response to drought stress, but the decreases were not obvious in Chl *a* content in L2 and in Chl *b* content in all transgenic lines. Therefore, the total chlorophyll content in transgenic lines L2, L4, and L6 decreased only by 10%, 18%, and 17%, respectively, compared with the decrease of 41% in the WT. The reduction in chlorophyll content in transgenic seedlings was not as high as that in WT seedlings, indicating that *TaPsb28* overexpression alleviated drought-induced injury by sustaining the chlorophyll content.

After 14 d of drought stress, the MDA content in the leaves of all plants was significantly higher than that in the normally watered control. The overexpression lines exhibited significantly lower MDA contents than WT (*p* < 0.05), which was decreased by 54%, 58%, and 49% for L2, L4, and L6 transgenic lines, respectively.

### 2.7. Overexpression of TaPsb28 Alleviates the Increases in ABA and Zeatin Contents but Improves the Increases in Cyanidin, Delphinidin, and PAs Contents under Drought Stress

**Figure 9:**
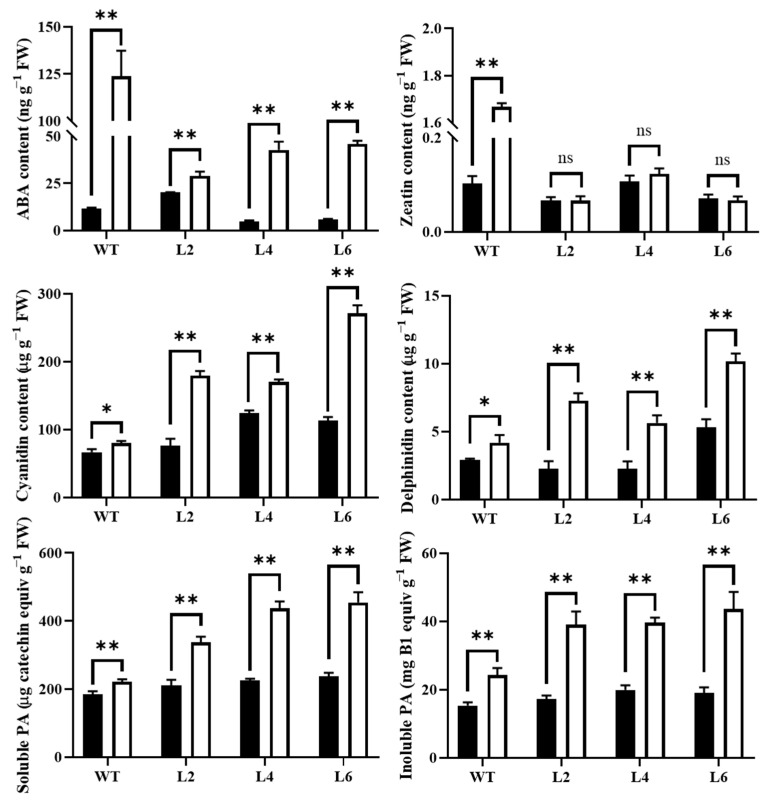
Abscisic acid (ABA), zeatin, cyanidin, delphinidin, and proanthocyanidin (PA) contents in leaves of *TaPsb28*-overexpressing *Arabidopsis* seedlings in response to drought stress. The black column represents the plants treated by normal watering, while the white column represents the plants subjected to drought stress by withholding irrigation for 14 days. Data are expressed as the means ± SDs (*n* = 3). “**” and “*” indicate statistically significant differences between the CK and drought treatments at *p* < 0.01 and 0.05, respectively, while “ns” represents nonsignificant differences between the two treatments at the level of *p* < 0.05.

When WT seedlings were subjected to drought stress, the ABA and zeatin contents in WT both increased significantly by 9.7- and 15.1-fold, respectively (Figure 9). Comparatively, the increase was obviously suppressed in transgenic plant lines. The ABA contents only increased by 0.4-, 8.1-, and 6.9-fold in L2, L4, and L6, respectively. No significant increase was observed in zeatin content.

*TaPsb28* was only located in the guard cell chloroplast around the stoma in overexpressing *Arabidopsis* lines. The main function of chloroplasts in plant guard cells is to regulate the level of sugar solutes in guard cells during stomatal opening [16]. *TaPsb28* gene overexpression improved stomatal closure to increase the water retention capacity, thus correspondingly alleviating the water loss rate and improving the survival rate. ABA is known to play an important role in plant resistance to drought stress by improving stomatal closure to decrease excess transpiration [27]. Induction of endogenous ABA accelerates ROS scavenging to decrease MDA content by improving the transcription of antioxidant enzyme-encoded genes [28]. However, in *TaPsb28*-overexpression lines, although stomatal closure was improved compared with that in WT plants, the increase in ABA content was suppressed. Similar inconsistent changes between increasing ABA content and stomatal closure under drought stress have also been reported in previous studies, such as those investigating *Cynophalla flexuosa* [29] and wheat [30]. 

Zeatin normally plays a negative role in drought tolerance because available evidence has shown the suppression of cytokinin signaling and biosynthesis under drought stress to improve drought resistance [31]. In this study, the increase in zeatin content in WT may inhibit the ABA effects on stomatal closure under drought stress [29]. This result was consistent with a previous report on peach seedlings that showed that the promoted biosynthesis of zeatin by exogenous application of silicon had an inhibitory effect on stomatal closure under drought stress [32]. Interestingly, in *TaPsb28*-overexpressing lines, the increase in zeatin under drought stress was restored to a nonsignificant level compared with that in normally watered plants. Therefore, we assume that the restored level of zeatin in *TaPsb28*-overexpressing plant lines may contribute to the removal of the inhibition of ABA function, thus promoting stomatal closure under comparatively reduced levels of ABA [33]. Similar to *Cynophalla flexuosa* [29], endogenous ABA leaf concentration had less of an effect on stomatal conductance, but the interaction between ABA and zeatin was more important in the stomatal aperture of *TaPsb28*-overexpression lines in response to drought stress.

Compared with WT, *TaPsb28* overexpression improved the increase in the anthocyanidin contents, including cyanidin, delphinidin, and the soluble and insoluble PAs. Anthocyanin accumulation was positively related to zeatin and ABA contents. Endogenous ABA has widely been accepted as being involved in the promotion of anthocyanin biosynthesis under drought stress in some plants, such as *Aristotelia chilensis* [34,35] and apples [36].

### 2.8. Overexpression of TaPsb28 Improved ChlG, DFR, and ANR Gene Transcription but Alleviated the Induction of RD22 Gene Transcription under Drought Stress

**Figure 10:**
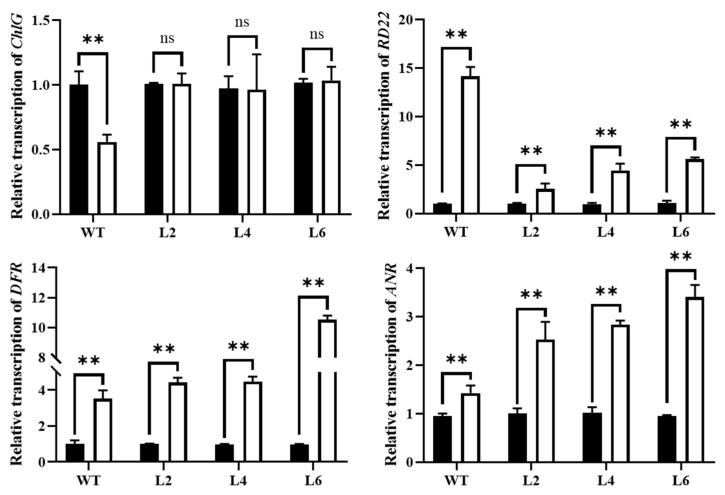
Relative transcriptions of *ChlG*, *DFR*, *ANR*, and *RF22* genes of *TaPsb28*-overexpressing *Arabidopsis* seedlings in response to drought stress. The black column represents the plants treated by normal watering, while the white column represents the plants subjected to drought stress by withholding irrigation for 14 days. Data are expressed as the means ± SDs (*n* = 3). “**” indicates statistically significant differences between the CK and drought treatments at *p* < 0.01, while “ns” represents nonsignificant differences between the two treatments at the level of *p* < 0.05.

As shown in Figure 10, the relative transcription of *ChlG* in WT plants decreased significantly by 44% in response to drought stress (*p* < 0.05). The transgenic lines under drought stress maintained almost the same level as the normally watered control (*p* > 0.05). ChlG catalyses the conversion of Chl *a* to Chl *b*, which plays an important role in chlorophyll synthesis and reutilization [14] and the synthesis of chlorophyll-binding proteins [13]. *ChlG* downregulation not only affects the overall content of chlorophyll but also affects the synthesis and stability of chlorophyll-binding proteins to improve the turnover of photosystems I and II [15]. When gene expression is blocked, leaves appear yellow-green [37]. A previous study showed that the induction of *ChlG* could stimulate chlorophyll biosynthesis and improve stress tolerance as shown by decreasing MDA content [38]. The maintenance of *ChlG* gene transcription in *TaPsb28*-overexpressing lines should be the reason why the decrease in chlorophyll content and leaf yellowing were inhibited compared with those in WT plants. 

Drought stress significantly increased the relative transcription levels of *RD22*, *DFR*, and *ANR* genes in the WT plants (*p* < 0.05). The transcriptional inductions of *DFR* and *ANR* genes were further improved in *TaPsb28*-overexpressing plant lines. However, the upregulation of *RD22* transcription was relieved in transgenic plant lines; only 1.4-, 3.6-, and 4.2-fold increases were determined in L2, L4, and L6 plants under drought stress, respectively, compared with a 13.2-fold increase in WT plants. Moreover, *RD22* transcript levels responded well to endogenous ABA content in this study. It is an ABA-inducible gene that subserves plant resistance to drought by inducing stomatal closure [39,40]. The inconsistency between the alleviation of ABA improving *RD22* gene transcription and the improvement of stomatal closure indicated that ABA may not perform the regulatory responsibility alone in *TaPsb28*-overexpression lines.

In WT plants, drought stress triggered the transcription of the *DFR* and *ANR* genes and then improved the synthesis of proanthocyanidin, cyanidin, and delphinidin. Some previous reports demonstrated an inhibitory effect of cytokinin on anthocyanin biosynthetic genes, including *DFR,* in *A. thaliana* [17,41], but other experiments have proposed a complex regulation of anthocyanin biosynthesis triggered by cytokinins [42]. The overexpression of *TaPsb28* in *Arabidopsis* improved the synthesis of anthocyanin by upregulating the transcript levels of the *DFR* and *ANR* genes. DFR is the key enzyme in the biosynthesis of anthocyanidins, such as cyanidin and delphinidin, while ANR is responsible for reducing anthocyanidins to proanthocyanidins [43]. Induced *ANR* transcription in transgenic *Populus ussuriensis* conferred drought tolerance by enhancing proanthocyanidin biosynthesis [44]. Therefore, endogenous zeatin production in *TaPsb28*-overexpressing plant lines was proposed to inhibit the accumulation of proanthocyanidins triggered by ABA because their accumulation only occurred when zeatin accumulation was relieved in transgenic plants.

Overall, the *TaPsb28* gene of wheat was cloned and characterized in this study. Drought stress dramatically improved its transcription. Overexpression of *TaPsb28* in *Arabidopsis* conferred drought tolerance, as exhibited by the increase in survival rate. Transgenic plants overexpressing *TaPsb28* maintained lower MDA content and higher chlorophyll content by the induction of *ChlG* transcription. Moreover, ABA and zeatin were proposed to cooperate to improve stomatal closure and proanthocyanidin accumulation in response to drought stress when *TasPsb28* was overexpressed. The understanding acquired through the research laid a foundation for further in-depth investigation of the function of *TaPsb28* in drought resistance in wheat. *TaPsb28* is a positive gene that can alleviate stress-induced damage and, therefore, has the potential for use as a candidate gene for the cultivation of drought-tolerant wheat cultivars.

## 3. Materials and Methods

### 3.1. Multiple Sequence Alignment and Bioinformatics Analysis of the TaPsb28 Gene

The wheat *TaPsb28* gene sequences were downloaded from the WheatlS database (https://urgi.versailles.inra.fr/wheatis, accessed on 10 October 2018), while homologous protein sequences were retrieved from the National Center for Biotechnology Information (NCBI). DNAMAN software was used to analyse the amino acid sequences of Psb28 family proteins under default parameters (*Lynnon* Biosoft). A phylogenetic tree was subsequently constructed using the neighbor-joining method with MEGA11 software with the following parameters: bootstrap test method (1000 replicates) and pairwise deletion [45].

### 3.2. Wheat Plant Materials, Growth Conditions and Drought Stress Treatment

The wheat cultivars Zhengmai 1860, Bainong 207, and Zhoumai 18 were selected as the plant materials because they are all widely planted cultivars in the main wheat-producing areas in Henan Province, China. All seeds were collected from Henan Academy of Agriculture Sciences. The seeds with consistently good germination were transferred from the culture dish to plastic pots with culture soil (organic nutrient soil: vermiculite, 3/1, *v/v*). The seedlings were cultivated by watering with 400 mL of water every 2 days until two leaves were fully expanded according to a previous report [46]. Then, three pots of seedlings were exposed to drought stress by ceasing watering, and the water loss rate of the soil was approximately 10.5% per day. Watering of the controls was continued. After 7 d of treatment, the leaves were sampled for subsequent analyses.

### 3.3. Vector Construction

The pYFPLT vector was used for subcellular localization of *TaPsb28* in maize mesophyll protoplasts. The full-length 546-bp cDNA of the *TaPsb28* gene was isolated from the leaves of Bainong 207 cultivar wheat seedlings using the primer set PYFPLT-*TaPsb28,* as shown in Table 1. After digestion by the XhoI and Hind II (NEB) enzymes, the CDS of the *TaPsb28* gene with corresponding restriction sites was inserted into linearized pYFPLT using the One-step Fast Cloning Kit (Shanghai Yisheng Biotechnology Co., Ltd., Yinchuan, China) The construct was introduced into competent *Escherichia coli* 2984, and the positive clones were then selected by plating on ampicillin-supplemented Luria-Bertani (LB, Land Bridge Technology Co., Ltd., Beijing, China) agar.

The pCAMBIA 1302 vector was used to generate transgenic *Arabidopsis*. The ORF sequence of *TaPsb28* was amplified from the pYFPLT-*TaPsb28* vector using the primer set 1302-*TaPsb28* (Table 1) and was then inserted into the pCAMBIA 1302 vector. Then, the construct was introduced into competent *Agrobacterium tumefaciens* GV3101 cells. The positive strains were selected by screening on LB agar supplemented with 50 μg/mL kanamycin and 25 μg/mL rifampicin. The accuracy of the constructed vector was verified by sequencing performed by Shangya Biological Co., Ltd., Zhengzhou, China.

### 3.4. Transformation of Arabidopsis

Excessive amount of the constructed pCAMBIA1302-*TaPsb28* was transformed into wild-type *Arabidopsis thaliana* (Columbia-0) by the floral dip method [49,50]. Transgenic overexpression lines were selected on MS medium containing 25 mg/L hygromycin supplementation, 2-week-old green seedlings of the T_2_ generation were confirmed by PCR and the product was validated by sequencing. The expression levels were identified by both qRT–PCR and Western blotting. The anti-GFP and anti-Actin antibodies used for Western blotting were purchased from Sangon Biotech (Shanghai) Co., Ltd., Shanghai, China.

### 3.5. Subcellular Localization of TaPsb28

The subcellular location of *TaPsb28* was examined both in mesophyll protoplasts from *Zea mays* (inbred Line B73) and in transgenic *Arabidopsis,* as described by Hotto et al. [51]. Mesophyll protoplasts were isolated from ~5 g of leaf tissue obtained from the middle of the second fully expanded leaves of 2- to 3-week-old seedling leaves. The integrity of the extracted mesophyll protoplasts was guaranteed by microscopy observation. Subsequently, pYFPLT-*TaPsb28* was transformed into protoplasts according to polyethylene glycol (PEG)-mediated transformation assays [52]. The transformed protoplasts were incubated at 25 °C for 12 h under dark conditions. The location of the fusion protein was examined for chloroplast autofluorescence and YFP fluorescence by a laser scanning confocal microscope (A1HD25, Nikon, Japan) at an excitation wavelength of 488 nm and instrumental magnification of ×40. For the subcellular location of *TaPsb28* in transgenic *Arabidopsis*, the 2-week-old leaves from T2 generation plants were retrieved for observation directly following the procedure described above.

### 3.6. Drought Stress in Arabidopsis

Homozygous T3 transgenic *Arabidopsis* seeds were used for further drought stress treatment. The germinated WT and transgenic seeds with uniform growth were transferred from MS medium to pots with mixed soil (vermiculite:humus = 1:1, 5–8 plants in each pot) and cultured in a greenhouse. After 2 weeks, the pots were divided into drought and control groups. For drought stress, *Arabidopsis* was treated by withholding irrigation for 14 days, and the phenotypes of stressed seedlings were observed. Then, the seedlings were rewatered for 5 days to assess the survival rate.

### 3.7. Measurement of Physiological Indices

To measure the water loss rate, leaves of *Arabidopsis* plants grown for 3 weeks were retrieved and immediately weighed to assess fresh weight (FW0). Leaves were then placed at 21 °C for air drying. The weight of the leaves (FW1) was measured every hour for 8 h. Each treatment was measured in triplicate, with 10 leaves for each measurement. The water loss rate was calculated as follows: (FW1 − FW0) × 100%/FW0_._

Three hundred milligrams of plant leaves were collected for chlorophyll content examination according to Porra et al. [53], with minor modification. A microplate reader (SpectraMax-i3x, Molecular Devices, USA) was used to evaluate the absorbance at 665 and 649 nm of the chlorophyll ethanal extract. The total chlorophyll content (C*_t_*), chlorophyll *a* (C*_a_*), and chlorophyll *b* (C*_b_*) were calculated using the following equations: C*_a_* = 13.95A_665_ − 6.88A_649_, C*_b_* = 24.96A_649_ − 7.32A_665_, C*_t_* = C*_a_* + C*_b_*, respectively. The malondialdehyde (MDA) content was determined using 1.0 g *Arabidopsis* leaves as described previously [7].

### 3.8. Measurement of Stomatal Aperture

The leaves were retrieved from the normally growing wide and transgenic *Arabidopsis*. The drought treatment was performed by placing the sliced leaves on a glass petri dish for 2 h to induce water loss. The lower epidermis of the leaves was torn off and then placed on a slide for stomatal aperture observation using a microscope. The control leaves without water loss treatment were observed directly after tearing off the lower epidermis. At least four leaves were observed for each treatment, and 25 stomata were recorded in each observation. ImageJ software (National Institutes of Health, Bethesda, MD, USA) was employed to evaluate the stomatal length and width, and the stomatal aperture was calculated as the ratio of width/length.

### 3.9. Quantification of Endogenous ABA and Zeatin

The endogenous ABA and zeatin contents were extracted from plant leaves using acetonitrile as the extraction solvent [54]. The samples were purified using the QuEChERS method and concentrated by a termovap sample concentrator (Shanghai Naai Instrument Co., Ltd., Shanghai, China). The separated hormones were quantified using an Agilent 1290 High-Performance Liquid Chromatograph system tandem AB Qtrap6500 mass spectrometer (Agilent Technologies, Inc., Santa Clara, CA, USA). A poroshell 120 SB-C18 reversed-phase column (2.7-µm particle size, 2.1 mm × 150 mm) was used as a separator. The samples to be tested were injected into the mobile phase by an automatic sampler. The ABA and zeatin contents were both calculated according to their individual curves generated from commercially purchased standards (Sigma-Aldrich, St. Louis, MO, USA).

### 3.10. Determination of Endogenous Cyanidin and Delphinidin Contents

The anthocyanin in leaves was extracted prior to HPLC analysis according to the method used by Cui et al. [43] with modifications. Half a gram of the leaf tissue was ground to a powder in liquid nitrogen and then extracted using 5 mL of buffer solution containing 70% methanol and 2% formic acid at 4 °C. After centrifugation at 12,000 rpm for 10 min, the supernatant was filtered with a 0.22-µm syringe filter prior to HPLC analysis using the same equipment as described above. The cyanidin and delphinidin contents were identified by comparing their individual retention time with standards (J&K Scientific LLC., Beijing, China) and quantified with standard curves.

### 3.11. Extraction and Quantification of Proanthocyanidin

Approximately 1 g of leaves was used for proanthocyanidin extraction with 5 mL of solution containing 70% acetone and 0.5% acetic acid as described by Yuan et al. [55]. The soluble proanthocyanidin content was determined using dimethylaminocinnamaldehyde (DMACA) with catechin standards. The same extracts were subjected to insoluble proanthocyanidin quantification using the butanol/HCl method by comparing the absorption at 550 nm based on a standard curve [56].

### 3.12. Total RNA Extraction, Reverse Transcription, and Real-Time PCR Quantification

Total RNA was extracted from 1 g of plant leaves using a TransZol Up Plus RNA kit (TransGen Biotech Co., Ltd., Beijing, China) according to the manufacturer’s recommendation. The first chain was synthesized by a HiScript II 1st Strand cDNA Synthesis Kit (Vazyme Biotech Co., Ltd., Nanjing, China). The cDNA was subjected to RT-qPCR using the StepOnePlus Real-time PCR system (Applied Biosystems, Foster City, CA, USA) and ChamQ Universal SYBR qPCR Master Mix (Vazyme Biotech Co., Ltd., Nanjing, China) according to the manufacturer’s instructions. All the primer sets are listed in Table 1. The relative expression levels of specific genes were calculated using the 2^−ΔΔCT^ method [57]. The amplification of Actin-1 and Actin-2 was used as an internal control for wheat and Arabidopsis, respectively.

### 3.13. Statistical Analysis

Each measurement was performed in triplicate independently, and the data are shown as the mean ± standard deviation (SD). The data were statistically analysed using SPSS software (version 18.0; SPSS, Chicago, IL, USA). The significance between treatments was compared using one-way ANOVA with the least significant difference (LSD) assessed at the level of *p* < 0.05 or *p* < 0.01.

## Figures and Tables

**Table 1:** List of primers used in this study.

Primer Name	Primer Sequence (5′-3′)	Purpose	Reference
*Psb28*-F	ATGCCGGCAGTGATGAAAGC	Clone of *TaPsb28* in wheat	This study
*Psb28*-R	GTTCTTGACGAACTGGAGGCC		
Actin-1-F	TGCTATCCTTCGTTTGGACCTT	qRT–PCR in wheat	[47]
Actin-1-R	AGCGGTTGTTGTGAGGGAGT		
Actin-2-F	CTTAACCCAAAGGCCAACAGA	qRT–PCR in *Arabidopsis*	This study
Actin-2-R	GCAAGGTCAAGACGGAGGAT		
qPCR*-Psb28–1-F*	AGCTGAATGCTTGTACCGAGT	qRT–PCR in wheat	This study
qPCR*-Psb28–1-R*	CGGTTTTCCTCATTACGTGCTT		
qPCR*-Psb28–2*-F	AAAGCCTGCAGTCCTCCTTC	qRT–PCR in *Arabidopsis*	This study
qPCR*-Psb28–2-R*	GTGCCCTGGATGAACTGGAT		
PYFPLT-*TaPsb28*-F	acaaatctatctctctcgagATGCCGGCAGTGATGAAAGC	Construction of subcellular vector	This study
PYFPLT-*TaPsb28*-R	ctacgcgtgagctcaagcttGTTCTTGACGAACTGGAGGCC		
1302-*TaPsb28*-F	cttgaccatggtagatctgactagtATGCCGGCAGTGATGAA	Construction of expression vector	This study
1302-*TaPsb28*-R	gaaaagttcttctcctttactagtGTTCTTGACGAACTGGAGGCC		
*RD22*-F	GCGATTCGTCTTCCTCTGAT	qRT–PCR	This study
*RD22*-R	CTCCGCCTTTACCTACTTGG		
*ChlG*-F	CTTCCGTCGGTTCTATG	qRT–PCR	This study
*ChlG*-F	CTTCCGTCGGTTCTATG		
*DFR*-F	CCTTATCACCGCGCTCTCT	qRT–PCR	[48]
*DFR*-R	TGTCCTTGTCTTATGATCGAGTAATGC		
*ANR*-F	AAGAAAACTGGACTGACGTTGAA	qRT–PCR	[48]
*ANR*-F	AACACCTTCGAGATTGGGTAAC		

## Data Availability

The data used and/or analyzed during the current study are available from the corresponding author on reasonable request.

## Abbreviation

ABA	Abscisic acid
ANR	Anthocyanin reductase
CDS	Coding sequence
ChlG	Chlorophyll synthase
CK	Control
DFR	Dihydroflavonol 4-reductase
LB	Luria-Bertani
MDA	Malondialdehyde
NCBI	National Center for Biotechnology Information
PSII	Photosystem II
ROS	Reactive oxygen species
SD	Standard deviation
WT	Wild-type
